# Agricultural extension and advisory services strategies during COVID‐19 lockdown

**DOI:** 10.1002/ael2.20056

**Published:** 2021-10-22

**Authors:** Anthony Baffoe‐Bonnie, David Tanner Martin, Frank Mrema

**Affiliations:** ^1^ Dep. of Agriculture Alcorn State Univ. Lorman MS 39096 USA

## Abstract

The COVID‐19 lockdown policies that began in 2020 caused an unprecedented shock to developing countries’ agricultural activities, especially those in sub‐Saharan Africa and South Asia. To reduce some of the impacts of COVID‐19 events, agricultural extension and advisory personnel created an avenue to assist farmers in these developing countries. However, since COVID‐19 protocols restricted public gatherings and close contact activities, agricultural extension activities had to be performed using unconventional ways such as mobile phones, radio, and television. This paper highlights some of the challenges agricultural extension and advisory service personnel encountered using these unconventional means of communication for their activities. We also present some solutions to these challenges that can enable policymakers to enhance agricultural extension activities performed unconventionally.

## Core Ideas

- COVID‐19 changed how agricultural extension services are performed in developing countries.
- Extension personnel used mobile phones, radio, and television to provide services.
- Agricultural extension personnel encountered many challenges using these unconventional ways.
- Telecommunication infrastructure needs to be developed in farming communities.
- Extension personnel should complement traditional face‐to‐face with online activities.

## 1 INTRODUCTION

In developing economies, agricultural activities are often done on a small scale; small farms make up about 84% of all farms (Lowder et al., 2016). However, the agricultural sector contributes substantially to the gross domestic product and employs a large proportion of the rural population in these countries (Baffoe‐Bonnie & Kostandini, 2019). Over the last decades, the contribution of agriculture to foreign exchange in developing countries has increased. It is estimated that developing countries share of total food exports increased from 31% in 1995 to 40.2% in 2018 (Glauber, 2020), and approximately 80% of people living in developing countries depend on agriculture as their main source of livelihood (Christiaensen et al., 2020). This shows that agriculture is crucial to developing countries economic growth. In addition, the agricultural sector improves human health in these countries by supplying nutritious food and reducing malnutrition and food insecurity.

The global pandemic (COVID‐19) has had an unprecedented effect on the economies of developing countries, affecting almost every facet of human activity, including agriculture. The lockdowns imposed by governments in developing countries to stop the spread of the virus prevented many farmers from accessing agricultural inputs and selling their produce. In some countries, farmers could not export their produce due to temporary export restrictions imposed to protect domestic demand (Glauber et al., 2020). As several new variants of the coronavirus continue to show up and COVID‐19 cases surge in developing countries, more disruptions will occur in agricultural activities. Therefore, there is a need to find ways to mitigate the COVID‐19 impacts on agricultural activities, especially in developing countries, where many people's livelihoods are dependent on agriculture. Agricultural extension and advisory services provide an avenue for farmers in these countries to learn about ways to reduce COVID‐19 impacts on their activities.

Extension and advisory services are critical to the agricultural sector during this time of uncertainty. This is because farmers need to know about ways to overcome some of the challenges created by the pandemic and prevent future disruptions in their activities. Also, they need to know about programs that can assist with their activities during this uncertain time. However, COVID‐19 protocols that restrict public gatherings and close contact activities have created challenges for agricultural and advisory extension personnel to reach farmers through conventional ways such as field‐day events, on‐farm demonstrations, and group training. A study by the Sasakawa Africa Association (SAA) in mid‐April 2020 showed that about 83% of African farmers did not receive any training from extension personnel when the lockdown started (SAA, 2020). As a result, agricultural extension personnel had to develop alternative means to reach farmers.

This paper highlights three main ways of communication adopted by agricultural extension and advisory services personnel in developing countries to assist small farmers during the COVID‐19 lockdown period and the challenges associated with each method. We also suggest potential solutions to address these challenges. The solutions would be helpful to agricultural stakeholders as many developing countries are seeing a surge in COVID‐19 cases and are finding ways to reform their agricultural sector. Finally, the proposed solutions can change the way agricultural extension and advisory services are delivered in developing countries.

## 2 MEANS OF COMMUNICATION BY EXTENSION AND ADVISORY SERVICES

### 2.1 Mobile phones

Mobile phones are an important tool in the agricultural sector of many developing countries. This is because they provide an opportunity to transfer knowledge and information to farmers at relatively less expense. They also allow farmers to build social relations among themselves and agricultural extension and advisory personnel. In addition, farmers who receive agricultural recommended practices via mobile phones are more likely to adopt these practices (Fabregas et al., 2019). The COVID‐19 pandemic increased the importance of mobile phones as a tool for agricultural extension and advisory services in most developing countries. In India, mobile phones became the only means farmers accessed information from agricultural extension workers during the lockdown (Dhulipala, 2020). Mobile phone communication applications such as WhatsApp (Facebook Group) were used by agricultural extension personnel to send messages, voice recordings, and short videos to farmers. For example, the Kenya Cereal Enhancement Climate‐Resilient Agricultural Livelihoods Window (KCEP‐CRAL) program used WhatsApp to provide agricultural extension services to farmers in Kenya (Even & Nyathi, 2020). These mobile phone applications helped agricultural extension workers to connect with farmers during the pandemic lockdown. In China, agricultural extension agents in Caohu provided information to farmers via WeChat (Tencent Holdings) during the pandemic (FAO, 2020b).

**Core Ideas**

- COVID‐19 changed how agricultural extension services are performed in developing countries.
- Extension personnel used mobile phones, radio, and television to provide services.
- Agricultural extension personnel encountered many challenges using these unconventional ways.
- Telecommunication infrastructure needs to be developed in farming communities.
- Extension personnel should complement traditional face‐to‐face with online activities.

One main challenge of using mobile phones to deliver agricultural extension services during the COVID‐19 lockdown was that most growers were not conversant with mobile applications like WhatsApp. The success of agricultural extension officers using mobile phones to reach farmers was limited because some agricultural producers could not use this technology (Joseph & Barry, 2021). This shows that farmers in developing countries do not use mobile phones beyond calling or receiving calls. According to Alvi et al. (2021), only 3% of women farmers in Gujarat, a western state in India, used mobile phones to make digital payments during the lockdown period. Another challenge was the limited access to Internet services in many agricultural communities. This made it difficult for farmers to rely on mobile phone applications to receive agricultural information and advice. This challenge affected both farmers and agricultural extension personnel since agricultural extension personnel are often located in rural areas with no Internet coverage or limited network. Hence, mobile phone and mobile applications‐based extension services were not conducive to the work of either farmers or extension personnel.

### 2.2 Radio

Radio is one of the oldest technologies and most trusted sources of information in most developing countries. Radio plays a vital role in agricultural extension, especially in communities with no Internet services and electricity. It offers communities the opportunity to access information, education, and entertainment (Nakabugu, 2001). The COVID‐19 lockdowns and social distance protocols initiated more efforts by many agricultural extension agencies to work with local radio networks to reach farmers. This collaborative effort allowed extension personnel to reach communities that were often cut off from their services. It also allowed information to be shared equally between men and women in some countries. During the lockdown, radio talk shows were organized to educate and inform farmers on good agronomic practices in many developing countries. In Uganda, for example, radio talk shows provided farmers with information on food hygiene and good farming practices (SAA, 2020). Farmers also received information on market prices and markets operating during the lockdown through these radio talk shows. In addition, talk shows were used to provide training for farmers. This was evident in Somalia, where the Food and Agriculture Organization (FAO) used radio to train farmers on good agronomic practices, livestock, nutrition, and fishery during the height of the pandemic and upsurge of the desert locust (FAO, 2020c). The training was intended to assist smallholder farmers in expanding their business and also becoming more resilient to changes in the agricultural sector.

Although radio offered an opportunity for agricultural extension personnel to continually reach farmers during the lockdown period, some challenges were encountered using radio talks to deliver extension services. First, growers had a limited time within the period assigned for the phone‐in sessions to ask questions that were relevant to their situation, and extension personnel could not answer all the questions within the allotted time. Hence, some farmers did not get solutions to their problems during the lockdown. Second, farmers living in areas with poor radio and phone networks had a comparative disadvantage in getting the needed information. These farmers could not tune into the radio talk shows or phone in to ask questions. This made it difficult for farmers in remote areas to receive agricultural extension advice via radio during the lockdown. Finally, choosing the right radio station for the agricultural extension was a challenge since it is difficult to know farmers’ favorite radio stations. Farmers prefer to listen to a radio station base on whether they understand the language or the alternatives available in their geographical areas. Therefore, many extension personnel did not know whether their targeted audiences were receiving their messages.

### 2.3 Television

Although television is expensive and less common among rural communities in developing countries, it is vital in disseminating information to farmers. Therefore, the rapid spread of television stations in developing countries allowed agricultural extension personnel to educate farmers during the COVID‐19 lockdown through television programs. Television programs were used to disseminate information on management practices, weather, and the location of agricultural inputs and output markets to farmers in Malawi (Even & Nyathi, 2020). Also, agricultural experts developed television programs to educate both farmers and nonfarmers on how to grow their crops. In Gabon, a television program presented by FAO experts and the National Federation of Agricultural Processors demonstrated how to cultivate vegetables (FAO, 2020a). Compared with radio, television provided farmers the benefit of seeing agricultural experts’ demonstrations, which was more helpful for the farmers.

However, like radio, television‐based agricultural extension services faced some challenges during the lockdown. For example, agricultural extension personnel needed to design interesting programs and tailor programs to address the specific and immediate needs of farmers. This is important because farmers need timely and accurate information concerning their challenges. Therefore, extension and advisory service personnel had to spend a long time designing programs to attract farmers. Another challenge extension officers encountered was that some farmers did not have access to electricity. Unlike radio, farmers need electricity to power their televisions. However, in many developing countries, electricity is rationed or unavailable in most remote or rural areas. It is estimated that about one billion people, mostly living in sub‐Saharan Africa and South Asia, do not have access to electricity (World Bank, 2018). Hence, television‐based extension services were not a good substitute for traditional face‐to‐face agricultural extensions in some developing countries, especially sub‐Saharan Africa and South Asia.

## 3 POTENTIAL SOLUTIONS TO CHALLENGES

As the COVID‐19 pandemic has increased mobile phone and mobile application‐based agricultural extension and advisory services (Payne, 2021), telecommunication infrastructure needs to be developed in rural communities to enhance the digitization of farming activities. Developing countries can achieve this through a strong partnership between governments, agricultural development agencies such as the United States Agency for International Development (USAID), agricultural stakeholders, and private telecommunication organizations. The partnership can help reduce the telecommunication charges for people in rural communities and increase the adoption of information and communication technologies (ICTs) for agricultural activities in rural communities. Low telecommunication charges can also help agricultural extension and advisory personnel to use Short Message Service (SMS) in areas with no Internet services or with farmers who do not have smartphones. However, education on the digitization of agricultural activities should continue to be promoted in these areas. This will encourage farmers to learn some of the technical skills needed for digital transformation (Payne, 2021).

Radio and television shows should also be tailored to the needs of farmers in the communities. Agricultural extension personnel can work with farmers cooperatives to know members’ needs and organize radio or television programs that address those needs. This will reduce the disconnect between the information presented during the talk shows and the information the farmers need. Additionally, agricultural extension and advisory personnel should target radio stations with a broader audience when using radio talk shows. This can help extension personnel to reach farmers in remote areas as well as reach a more diverse group, especially women and young farmers.

Finally, agricultural extension and advisory personnel should complement traditional face‐to‐face interactions with online or virtual activities when possible. This allows farmers to adapt to these ways of delivering extension services and learn more about the technologies. It is also important that the infrastructural base of these communities be expanded, especially access to electricity and good road networks. Providing these infrastructures will enhance agricultural extension activities and help develop agriculture by creating market access for rural farmers.

## 4 CONCLUSION

The COVID‐19 pandemic has affected many activities, including agricultural extension and advisory services. Three primary means of communication were adopted by agricultural extension and advisory personnel in developing countries to connect with farmers during the lockdown period: through mobile phones and mobile applications, radio talk shows, and television programs. The benefits of using these means of communication to deliver extension services were not fully realized due to challenges such as limited access to Internet services, difficulty in using mobile phone applications, and poor infrastructure. To answer these challenges, there is a need to develop the infrastructure base of most agricultural communities in developing countries and complement unconventional means of delivering agricultural extension services with traditional face‐to‐face extension activities during normal times. This will help speed up the digitalization of the agricultural sector of developing countries.

## AUTHOR CONTRIBUTIONS

Anthony Baffoe‐Bonnie: Conceptualization; Writing‐original draft; Writing‐review & editing. David Tanner Martin: Writing‐original draft. Frank Mrema: Conceptualization.

## CONFLICT OF INTEREST

The authors declare no conflict of interest.
